# Physiological effects of spirulina supplementation during lactate threshold exercise at simulated altitude (2,500 m): a randomized controlled trial

**DOI:** 10.1080/15502783.2025.2498484

**Published:** 2025-05-01

**Authors:** Tom Gurney, James Brouner, Owen Spendiff

**Affiliations:** a Kingston University, School of Life Sciences, Kingston upon Thames, England; b University College London, Division of Surgery & Interventional Science, London, UK

**Keywords:** Altitude, algae, cycling, lactate, heart rate

## Abstract

**Background:**

Existing literature indicates that spirulina supplementation can improve a variety of blood morphological parameters (such as hemoglobin) in healthy and clinical populations. Given the importance of hemoglobin for endurance exercise, particularly at altitude, this study aimed to investigate whether spirulina supplementation can improve blood morphological parameters in healthy cyclists and positively influence physiological variables when completing a lactate threshold test at simulated moderate altitude (2,500 m).

**Methods:**

Twenty (18 male; 2 female) healthy participants (Mean  ±  SD; Age 37   ±   9 years, Stature 181   ±   6 cm, Mass 81   ±   9 kg, $\dot{V}$O_2max_ 51.8   ±   7.8 ml·kg-1·min-1) ingested 6 g/day of spirulina or placebo for 3-weeks in a double-blinded randomized counter-balanced cross-over design, then completed a lactate threshold test at simulated moderate altitude, with a 14-day washout period between trials.

**Results:**

Following spirulina supplementation, heart rate at the lactate threshold was significantly lower in comparison to placebo supplementation (spirulina = 152   ±   11 b.min −1 vs. placebo = 155   ±   12 b.min −1, *p* < 0.05). No other physiological variables (Watts, RER, RPE, VO_2_) were significantly different (*p* > 0.05) at the calculated threshold, or during the first four lower stages. Red Cell Distribution Width significantly increased following spirulina supplementation in comparison to placebo (spirulina = 13.3   ±   0.9 % vs. placebo = 12.5   ±   0.7 %, *p* < 0.05). Plateletcrit significantly decreased following spirulina supplementation (spirulina = 0.288   ±   0.032 vs. placebo = 0.293   ±   0.050, *p* < 0.05). No other blood morphological parameters changed (*p* > 0.05).

**Conclusion:**

In conclusion, three weeks of spirulina supplementation at 6 g/day reduced heart rate during a lactate threshold test at a simulated moderate altitude (2,500 m), but did not produce additional physiological or hematological benefits.

## 1. Introduction

Although most endurance cycling events occur at sea level or low altitude, competing and training at moderate altitude (2000 m  −  3000 m, as defined by the International Olympic committee [1]) are becoming increasingly common [2]. At these elevations, the partial pressure of oxygen declines despite a constant fraction of inspired oxygen (FiO₂ = 20.9%), reducing the effective FiO₂ to approximately 14.8–16.7% [3]. This moderately hypoxic environment triggers well-known physiological challenges for athletes, including reduced oxygen saturation and impaired delivery to muscles, which can compromise key endurance parameters such as $\dot{V}$O_2max_, lactate threshold (LT), and power output [4,5].

To negate negative effects on performance, athletes try a variety of training acclimation strategies in preparation for a specific event. The predominant adaptive response athletes pursue from altitude training is an enhanced stimulation of erythropoietin (EPO) which sequentially drives an increase in Hb circulation [4,5]. The positive effects Hb exert on endurance performance are well reported [6,7], briefly, trivial increases in Hb have been associated with improving oxygen transport to the working muscles which therefore could improve maximal aerobic oxidative capacity [8], especially at altitude. It is however important to note that $\dot{V}$O_2max_ is not the only determinant of endurance performance [9], Lactate Threshold is also a key determinant [10]. As increases in Hb can affect the transport capacity of blood parameters like bicarbonate, lactate, and hydrogen ions during exercise [9], enhancements in this mechanism have also been proposed to support improved endurance performance [7,11,12].

For athletes who are seeking to improve performance at low-moderate altitude terrains, altitude training can be desirable, but costly and time-consuming. It is logical to assume that any alternative strategy that may stimulate blood morphological parameters would be an advantage. Nutritional strategies for example, at moderate altitudes, are still relatively unexplored [5].

Spirulina supplementation to date has demonstrated an array of positive effects on blood morphological capabilities in anemic populations, and most recently healthy populations, where it has been suggested that the iron content ( ~ 28 mg per 100 g) may stimulate erythropoiesis [11–14]. While some studies examining spirulina supplementation have reported improvements in endurance capacity, oxygen utilization, and markers of fatigue [13–18], some have found minimal or inconsistent effects [19–24]. Nevertheless, previous work has demonstrated that a 6 g/day 3-week spirulina supplementation period exerted positive effects on submaximal endurance markers (lactate & HR), primarily attributed to the 6.4% Hb increase as measured via the HemoCue 201+ [13]. Given the apparent link between Hb improvements and exercise performance at altitude, further research into spirulina and its potential applications is strongly warranted. However, little is still known about the underlying mechanisms in healthy populations or the consistency of these effects. The HemoCue 201+ measures only a single blood morphological parameter (Hb); therefore, a full blood count is necessary to determine which other blood parameters may be influenced by spirulina supplementation. To the best of our knowledge, only one study to date has conducted a full blood count analysis when investigating the effects of spirulina supplementation in healthy athlete populations [12].

Critically, whether and how changes in blood count parameters influence exercise performance at altitude following spirulina supplementation remain unexplored. Given the growing popularity of moderate-altitude events and the widespread use of LT markers by athletes and coaches to monitor training and performance, further investigation is warranted. Therefore, the primary aim of this study was to examine the submaximal physiological effects of three weeks of spirulina supplementation (6 g/day) during a LT test at moderate altitude (2,500 m). A secondary aim was to assess the effects of spirulina supplementation on full blood counts. It was hypothesized that spirulina supplementation would positively impact blood morphological parameters, thereby improving physiological responses during a lactate threshold test.

## 2. Material and methods

### 2.1. Participants

Based on the positive submaximal HR results from a previous study on spirulina supplementation [13], a priori power analysis was conducted using G*Power® (version 3.1.9.7; Universität Kiel, Kiel, Germany) [25]. The analysis indicated that a minimum sample size of six participants was required to detect a medium effect (0.73) of spirulina supplementation, with an alpha level of 0.05 and a power of 0.90. Twenty-three participants were consequently initially recruited for the study and provided written informed consent. Participants were required to be training at least 4–5 hours per week and have a minimum of two years of cycling experience. Exclusion criteria also included current smoking, known allergies to mold or algae, and any history of cardiovascular disease. Three participants withdrew for personal reasons, resulting in a final sample of 20 healthy participants (18 male, 2 female) (Mean ± SD: Age 37  ±  9 years, Stature 181  ±  6 cm, Mass 81  ±  9 kg, $\dot{V}$O2max 51.8  ±  7.8 ml·kg − 1 ·min − 1). The Faculty of Science, Engineering and Computing Ethics Committee at Kingston University London approved the study (1819.056.1) in accordance with the Declaration of Helsinki. The study followed the CONSORT and Good Clinical Practice guidelines. CONSORT diagram can be found in Supplementary File 1.

### 2.2. Study design

In a double-blind randomized crossover design, participants were required to visit the laboratory on three separate occasions, at the same time of day, 2 hours postprandial and were asked to refrain from heavy exercise 48 hours before each visit. The first visit comprised baseline anthropometric measurements and included a $\dot{V}$O_2max_ test. Additionally, a familiarization of the lactate threshold test at simulated altitude occurred during visit 1, see below for exercise protocols. Throughout the intervention, participants were instructed to avoid taking any other supplements, maintain their regular training regime/km cycles (Table 1), and record a 48-hour food and exercise diary before the first session, which was replicated before each subsequent visit. Two hours before arriving at the laboratory participants were asked to consume a standardized small snack (1 slice of brown toast, 5 g butter, 200 ml orange juice), and adherence to the standardized snack and 48-hour diary was 100%.

**Table 1.** Self-reported weekly KM cycled before each visit.

	Spirulina Visit	Placebo Visit	P Value
Self-reported weekly KM cycled	424 ± 93 km	408 ± 78 km	0.753

Participants were then randomly allocated to either spirulina (Indigo Herbs Limited; see supplementary file 2 for nutritional composition) or placebo (microcrystalline cellulose) in a counter-balanced design, akin to block randomization to ensure equal group sizes. They were instructed to ingest 6 g (12 capsules: 4 with breakfast, 4 with lunch, 4 with dinner) each day for 3 weeks. An independent laboratory technician generated the randomization sequence using a computer-based random number generator in Microsoft Excel and assigned participants to conditions accordingly. All capsules were visually identical and packaged into 21 small, sealed bags  −  1 for each day of the 3-week supplementation period. Capsule bags were coded with alphanumeric identifiers (e.g. “A1,” “B2,” etc.) that bore no indication of group assignment. These codes were linked to the randomization list and securely stored by the technician until data collection was complete. Allocation concealment and double blinding were maintained throughout the study. No participants reported any visual or taste differences or gastrointestinal issues after each supplement and could not guess which supplement they were taking following exit questionnaires administered 1-week following each participant’s last visit. The morning after the 3-week supplementation period, participants reported to the laboratories to begin their lactate threshold test at a simulated altitude (2500 m).

### 2.3. Exercise protocols

#### Baseline measurements & $\dot{V}$O_2max_ test

2.3.1.

During the first visit, and at the beginning of each subsequent visit, body mass (kg) and a 20-µL capillary blood sample for resting lactate and glucose (g/L) were taken from the finger for analysis (Biosen C-Line Sport, EKF GmbH, Germany). Thereafter, participants were asked to adjust the Monark 894E Peak Bike (Monark Exercise AB, S-432 82 Varberg, Sweden) to their preferred saddle height, which was recorded and replicated for each subsequent visit.

The $\dot{V}$O_2max_ data collection methods and equipment replicated the previous work by Gurney and colleagues [13]. Briefly, the test comprised an initial 3-minute warm-up with no resistance (0 W) at a cadence of 60–70 revolutions per minute (r .min −1). The test started at an initial power of 120 W and participants were asked to cycle consistently throughout the test at their preferred r min −1 (to be replicated for each visit). The load then increased by 25 W per minute until volitional exhaustion, or the test was terminated due to the r min −1 dropping by more than 10 r .min −1 for more than 20 seconds. Respiratory variables were collected continuously using the Vyntus CPX (Vyaire Medical GmbH, Germany) and HR data using a Polar H9 strap (Polar Electro Oy, Kempele, Finland). The $\dot{V}$O_2max_ was determined by the highest $\dot{V}$O_2_ value recorded from the 15-second averages. A 5-minute active recovery (25 W) was then given to participants.

#### 2.3.2. Lactate threshold test

To assess ventilatory variables at simulated altitude, the Vyntus CPX device was calibrated as per the manufacturer’s instructions in the High/Low FiO2 function – the software applies the 1987 Eschenbacher transformation [26] for the calculations when manipulating FiO2, as the Haldane transformation [27] has been reported to provide unreliable data [28]. Participants sat motionless on the bike whilst the mask and HR strap were fitted appropriately. The Everest Summit Hypoxic Generator (The Altitude Centre, UK) was then turned set to 2500 m with airflow tubes pumping a constant 15% FiO2 concentration into a 200 L Douglas bag (Cranlea & Co Ltd., Birmingham, UK) reservoir, large tubing was connected to a Y-valve which was attached to the Vyntus CPX volume/gas sensor and consequently the mask. The Vyntus CPX support helmet was placed onto each participant’s head, which supported the Y-Valve’s weight and volume/gas sensor, an essential step to ensure the mouthpiece does not slip out during testing. To test the reliability of this altitude system and exercise protocol, 5 participants completed two separate LT tests at the same time of day 1-week apart. The reliability of the physiological responses the LT are expressed as CV%: HR = 1.9%, Power = 2.9%, RPE = 5.1%, $\dot{V}$O_2_ = 2.7%. Further details can be seen in Supplementary File 3 and photos of the methodological set of the equipment can be seen in Supplementary File 4.

Once participants were connected to the Vyntus mouthpiece they sat at rest for 15 minutes before exercise to allow for their physiological variables (VO_2_, respiratory exchange ratio (RER), Hb saturation) to adjust and settle to equilibrium, as per the user manual. Once equilibrium was reached, an incremental LT test was performed (replicated from previous research [13,29]). Briefly, each participant started at an intensity relative to their fitness (in the current study this ranged from 80 W  −  200W) and their preferred r .min −1. The load was manually adjusted onto the Monark 894E Peak Bike where the incremental test protocol comprised increasing 25 W every 4-minutes until volitional exhaustion, or until the test was terminated due to either the r min −1 dropping for more than 20-seconds or if the SpO_2_ dropped below 80%. Capillary blood samples, RPE, SpO_2_, and HR were collected at rest and in the final 30 seconds of each 4-minute stage. The LT was calculated using the Dmax method in Lactate E software [30]. Power output, VO_2_, and HR at the calculated LT were compared for each condition.

### 2.4. Full blood count collection

The Yumizen H500 (HORIBA UK Limited, Northampton, UK) hematological analyzer was employed to conduct full blood count analysis following both supplementation periods. Capillary blood samples (50 µL) were collected upon arrival to the laboratory in microcuvettes before being placed immediately in the Yumizen H500 for analysis. To test the reliability of the Yumizen H500, 5 participants completed two separate tests at the same time of day 1-week apart. The reliability, expressed as CV%, for each variable can be found in Supplementary File 5.

### 2.5. Statistical analysis

Data are presented as mean ± SD. All statistical analyses were performed using IBM SPSS version 28 for Windows. All datasets were analyzed for normality using Shapiro – Wilk, while Mauchly’s test of sphericity was employed to establish any potential violations. Any violation of sphericity was corrected using the value from the Greenhouse – Geisser. Statistical significance alpha level was set at ≤ 0.05. Effect sizes (ES) calculated using partial eta squared SPSS output, observed power, and 95% confidence intervals (CIs) were used where appropriate. All variables during the first four stages of the LT test were analyzed using a 2-way within-subjects repeated-measures ANOVA, using a Bonferroni correction for multiple comparisons to determine any differences. Heart rate, power output, and VO2 at the calculated LT and all cell counter variables were compared using a paired sample t-test.

## 3. Results

### 3.1. At the calculated lactate threshold (Dmax)

**Figure 1.**
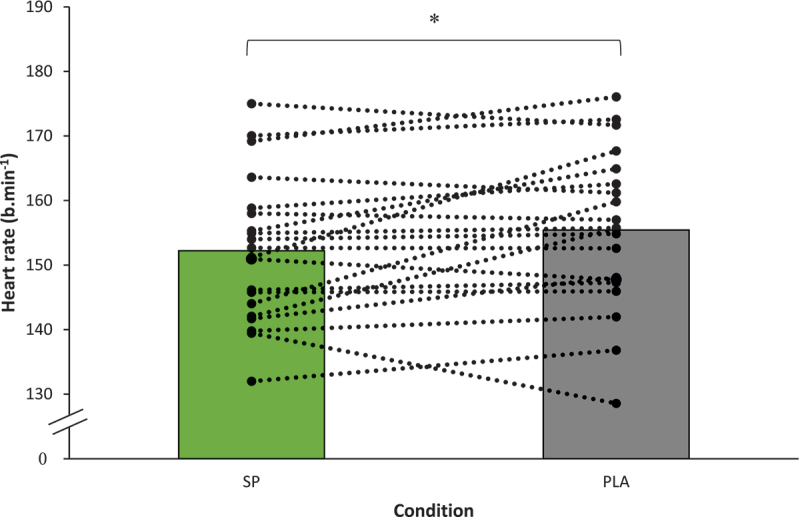
Heart rate at the calculated lactate threshold following the 6 g/day 3-week spirulina or placebo supplementation.

At the calculated threshold, power (spirulina = 190  ±  39 Watts vs. placebo = 190  ±  37 Watts), VO_2_ (spirulina = 33.4 mL/min/kg ±6.2 vs. placebo = 32.7  ±  6.0 mL/min/kg), RPE (spirulina = 12.9  ±  1.3 vs. placebo = 13.2  ±  1.1) and RER (spirulina = 1.01  ±  0.03 vs. placebo = 1.00  ±  0.04) were not significantly different (*p* = 0.857, *p* = 0.352, *p* = 0.160, and *p* = 0.292, respectively) following either spirulina or placebo ingestion. Whereas following spirulina supplementation, there was a small but significant (*p* = 0.049, Cohen’s d = 0.47, 95% CI:  − 6.37 –  − 0.02) reduction in HR at the calculated threshold (spirulina = 152  ±  11 b.min −1 vs. placebo = 155  ±  12 b.min −1), see Figure 1.

### 3.2. During the first four stages

#### 3.2.1. Main effects

There were no significant main effects (*p* > 0.05) for HR, lactate, SpO_2_, RPE, VO_2_ following spirulina or placebo ingestion.

#### 3.2.2. Time effects

A significant effect (*p* < 0.001) for time was detected in all variables, with both conditions demonstrating increases at every stage for HR, lactate, RPE, VO_2_ and decreases at every stage for SpO_2_ (*p* < 0.05).

#### 3.2.3. Interaction effects

There were no condition*time interaction effects detected in all variables (HR, lactate, SpO_2_, RPE, VO_2_) following spirulina or placebo ingestion (*p* > 0.05).

### 3.3. Cell counter

Paired sample T-Tests revealed that following spirulina supplementation RDW significantly increased (*p* = 0.001, Cohen’s d = 0.64, 95% CI = 0.42–1.16) and Plateletcrit significantly decreased (*p* = 0.023, Cohen’s d = 0.11, 95% CI:  − 0.088 –  − 0.007), though these variables were both still within their normal ranges [31]. There were no significant changes in all other variables from the cell counter (*p* > 0.05), see Table 2.

**Table 2.** Cell counter variables following 6 g/day 3-week spirulina or placebo supplementation (*n* = 13). Bold indicates *p* < 0.05.

	Spirulina ± SD	Placebo ± SD	P Value
Red Blood Cell (10 6/µL)	4.7 ± 0.4	4.7 ± 0.5	.844
Haemoglobin (g/dL)	148.8 ± 8.8	148.2 ± 11.3	.795
Haematocrit (%)	43.0 ± 2.8	43.1 ± 3.7	.956
Mean Corpuscular Volume (µm 3)	91.3 ± 3.0	91.2 ± 3.3	.865
Mean Corpuscular Haemoglobin (pg)	36.0 ± 16.9	31.5 ± 1.7	.347
Mean Corpuscular Haemoglobin Concentration (g/dL)	34.2 ± 0.8	34.5 ± 0.8	.082
Red Cell Distribution Width (%)	13.3 ± 0.9	12.5 ± 0.7	.001
Red Cell Distribution Width Standard Deviation (µm 3)	43.7 ± 2.5	42.1 ± 2.2	.002
Platelets (10 3/µL)	210.0 ± 34. 8	217.1 ± 44.9	.565
Plateletcrit (%)	0.288 ± 0.032	0.293 ± 0.050	.023
Mean Platelet Volume (µm 3)	8.9 ± 0.7	8.7 ± 0.9	.105
Platelet Distribution Width (µm 3)	14.5 ± 2.2	13.9 ± 2.2	.098
Platelets – Large Cell Count (10 3/µL)	54.9 ± 14.4	55.9 ± 23.0	.836
Platelet Large Cell Ratio (%)	26.9 ± 6.1	25.5 ± 7.6	.161
White Blood Cells (10 3/µL)	6.0 ± 1.40	5.7 ± 1.46	.462
Neutrophils (10 3/µL)	3.6 ± 1.15	3.2 ± 1.21	.218
Lymphocytes (10 3/µL)	1.6 ± 0.49	1.7 ± 0.67	.547
Monocytes (10 3/µL)	0.51 ± 0.21	0.45 ± 0.21	.148
Eosinophils (10 3/µL)	0.15 ± 0.11	0.13 ± 0.08	.274
Basophils (10 3/µL)	0.06 ± 0.02	0.06 ± 0.04	.907
Large Immature cells (10 3/µL)	0.02 ± 0.02	0.01 ± 0.01	.367

## 4. Discussion

Spirulina supplementation (6 g/day for three weeks) led to a small but significant reduction in heart rate at lactate threshold (LT) at moderate altitude (2500 m) (Figure 1) but did not improve other physiological variables. Additionally, supplementation influenced red cell distribution width and plateletcrit. To our knowledge, this study is the first to examine spirulina’s effects on LT physiological responses at moderate altitude and only the second to assess full blood count levels post-supplementation in healthy athletes. Given the growing interest in nutritional strategies to enhance endurance performance at altitude, these findings provide novel insights into spirulina’s potential physiological effects, offering practical implications for athletes and coaches seeking to optimize training and adaptation in hypoxic conditions. Existing literature on the efficacy of spirulina supplementation for exercise performance has yielded equivocal results with some research groups reporting positive effects [13–18,32] and others reporting no effect [19,22,24]. For example, employing the same supplementation period and dose, spirulina did not influence physiological responses during a LT at sea level [13], yet at moderate altitude in this study our findings indicate that spirulina may provide a beneficial effect on lowering HR at the LT. Though true comparison of the results is hard due to both tests being completed in different conditions (24 hours after exhaustive exercise vs. moderate altitude in the present study). Despite this, a reduced HR at the LT may have practical implications for athletes when training or competing at moderate altitude. For example, a lower HR at a given submaximal workload can reflect improved cardiovascular efficiency, potentially indicating that the same relative intensity requires less physiological strain. Over time this can allow athletes to sustain higher workload with less fatigue. However, while the HR findings in the current study were significant (*p* = 0.049), the clinical relevance of a  ~  3 bpm reduction remains uncertain, particularly given the small-moderate effect size (Cohen’s d = 0.47) and 95% CI bordering on zero (95% CI:  −6.37 –  −0.02).

During acute altitude exposure, one compensatory mechanism is an increased sympathetic activity and vagal withdrawal which drives an increase in HR at a given level of exercise [33,34], and will in turn lower stroke volume due to a drop in left ventricle filling pressure. Consequently, submaximal cardiac output is increased [33]. Considering that the given level of exercise in both supplements was the same at the LT (spirulina = 190  ±  39 Watts vs. placebo = 190  ±  37 Watts, *p* > 0.05), it indicates that spirulina supplementation at altitude could hypothetically maintain stroke volume or alleviate the reliance on compensatory mechanisms during exercise at moderate altitude. Although we did not assess directly, this may support the notion that spirulina supplementation can contribute to NO availability in the aorta [35,36]. Indeed, at sea level, spirulina supplementation has already been reported to improve the HR response during/following exercise [13,14,17], supposedly due to spirulina supplementation improving oxidative capacity [17,18,37]. Kalpana and colleagues [17] discussed that this effect was attributable to improvements in fitness, cellular signaling function of ROS, and antioxidant status. In previous work [13,14] it was speculated that improvements in Hb following spirulina supplementation contribute to lower homeostatic disturbances during exercise (lactate & HR). However, our results contradict this hypothesis as a lower HR at LT was observed, yet no improvement was seen in Hb indicating that the improvements in heart rate following spirulina supplementation cannot be solely attributed to increases in hemoglobin levels, rather it could be the combination of multiple constituents found in spirulina acting synergistically either directly or indirectly [38]. For example, the cysteine content found in spirulina can contribute to optimal muscle function [39]; the phycocyanin can contribute to NO availability in the aorta [35,36]; the multiple vitamins may contribute to the correction of nutrient deficiencies [38] which is widely understood to be optimal for general health and performance [40]; the antioxidants may prevent exercise induced muscle damage [23].

It is important to note that although there was a positive HR response at the calculated threshold, this may not necessarily be translatable to improvements in performance. For instance, there were no other improvements when comparing the rest of the physiological variables (power output, VO_2_, RER, RPE) at the calculated threshold. Given that power has been reported to be a determinant of performance [41] and therefore a key performance indicator in cycling, this would be considered the most pertinent variable an athlete might want to improve. Yet mean power output was the same (190 Watts) after ingesting both supplements. Further analysis of the variables during the first four lower stages of the test also confirmed that spirulina supplementation is not efficacious at simulated altitude. During these lower intensities, it is evident that the intensity may not be high enough (even at simulated altitude) for the requirement of an ergogenic aid. Despite this, the data from this study adds to the relatively limited body of literature, helping researchers identify the specific intensities and environments where spirulina supplementation may be most beneficial.

The key finding from the secondary aim was supplementation of spirulina did not improve Hb but significantly increased RDW, see Table 2. Red Cell Distribution Width is a measure to quantify the degree of variation between red blood cell size [42]. Accordingly, it may reflect abnormal red blood cell survival but also erythropoiesis [31]. The last decade has seen multiple reports demonstrating strong correlations between high RDW and all-cause morbidity [42]. The findings from the current study are consistent with the only other investigation that conducted full blood count analysis in humans after spirulina supplementation, where 12 sportsmen ingested 2.25 g/day for 14-days [12]. However, although RDW increased following spirulina supplementation, this increase was still within the normal range (11–15%, [31]). Interpretation of changes in RDW are complex given that RDW can be influenced by multiple factors which include age, physical activity behavior, hypertension, inflammation, and metabolic syndrome [43]. Indeed, even immediately after acute exercise (high intensity incremental running) RDW has been shown to increase [44]. To control for such covariates, 48-hour training logs were completed by participants in this study and compliance was 100%. There was also no significant difference in self-reported weekly reported km between each visit (Table 1).

Previous studies have not offered explanation or explored the underlying mechanisms behind increases in RDW following spirulina supplementation. We propose two conflicting hypotheses: one suggesting a beneficial effect and the other a potential negative impact. The first hypothesis is that an increase in RDW may indicate the early stages of iron mobilization or erythropoiesis, as greater variation in RBC size can occur when newly formed, larger RBCs mix with older, smaller ones. Alternatively, spirulina’s antioxidant properties may influence RBC lifespan, with some cells surviving longer while others turn over more rapidly, increasing size variability. However, without measurements of reticulocytes or ferritin, and in the absence of a rise in Hb, these explanations remain speculative and hypotheses based.

In addition, we observed changes in plateletcrit, which was higher in the placebo condition (Table 2). While this was a measured outcome, its physiological significance remains unclear. Some studies suggest that spirulina may possess mild anti-platelet or anti-coagulant properties, potentially due to its phycocyanin content, which has been linked to anti-inflammatory and vasodilatory effects [45,46]. Moreover, spirulina contains other compounds like calcium spirulan, which also exhibit anticoagulant properties by inhibiting thrombin [46]. However, we did not assess platelet function or coagulation markers in this study, and thus cannot confirm whether the observed plateletcrit reduction reflects such mechanisms. These interpretations should therefore be regarded as speculative hypotheses that warrant further investigation, rather than definitive conclusions drawn from the present data.

The second hypothesis is that changes in RDW may indicate a negative impact on vitamin B12 absorption. New emerging research now indicates that the vast majority of vitamin B12 in spirulina (between 72.6% and 97.9%) has been identified as “pseudo” vitamin B12 [47]. Pseudo vitamin B12 are biologically non-available for humans or animals (cannot be metabolized) [48], yet it competes with active vitamin B12 to bind with transcobalamin II (a carrier protein which is essential for the absorption, transport and cellular uptake of vitamin B12) [49,50]. Currently, the UK’s daily recommended vitamin B12 nutrient intake is 1.5 µg [51]. Though one group of vitamin B12 experts suggest this standardized approach is flawed as it does not account for individuals who are prone to developing vitamin B12 deficiencies (pregnant women, vegetarian and vegan populations), so the daily recommended intake should be individualized and be between 4–20 µg [52]. Using data from Van den Oever and Mayer [47], it is possible that participants in this study were consuming up to 9.8 µg of pseudo B12 per day, 8.3 µg over the current guidelines (though we did not measure this directly). It is therefore apparent and plausible to suggest that a substantial amount of pseudo vitamin B12 absorbed from spirulina supplementation can potentially disrupt vitamin B12 absorption and transport. Indeed, increases/elevated RDW levels have previously been strongly associated with vitamin B12 deficiencies [53], although this has been disputed to be the best (early) indicator [54]. In summary, although somewhat speculative and hypothesis driven, the relatively long and high spirulina supplementation period/dose employed in the current study may have inadvertently caused a brief disruption in vitamin B12 uptake/transport, as evidenced by a higher RDW. Such interpretation should come with caution as ultimately RDW, MVC, and Hb were still within normal ranges, we did not measure vitamin B12 directly, and developing a vitamin B12 deficiency can usually take months [55]. To support and improve this hypothesis in the future, vitamin B12 screening, or biomarkers associated with vitamin B12 (ferritin, transferrin), in participants should be conducted pre and post supplementation. Additionally, the (pseudo) vitamin B12 in the spirulina that was used should also be analyzed.

A major limitation of our study is the absence of baseline and post-intervention nutritional assessments, including key markers such as serum vitamin B12, ferritin, and dietary iron intake. Given that spirulina contains vitamin/pseudo-vitamin B12, iron, and various other antioxidant compounds, this omission critically impairs our ability to interpret the hematological outcomes, particularly the observed increase in RDW. Without these data, it is not possible to determine whether changes in blood parameters were driven by nutritional deficiencies at baseline, the bioavailability of spirulina-derived nutrients, or other physiological mechanisms. While we propose potential mechanisms involving B12-related pathways and red blood cell turnover, these remain speculative and should be regarded as hypotheses requiring further investigation. Future studies should incorporate comprehensive nutritional assessments to clarify the biological pathways involved. Secondly, while lactate threshold data is recognized as a determinant of athletic performance, access to proper lactate threshold testing is limited for most athletes, thereby restricting the applicability of our results. A time trial would have been a more effective and practical measure as it is a direct measure of exercise performance. A third key limitation is the use of microcrystalline cellulose as the placebo, which, while inert, does not nutritionally match spirulina. Without an iso-caloric, iso-nutrient comparator, it is not possible to determine whether the observed effects stem from spirulina itself or from its broader nutritional composition. Future research should incorporate nutrient-matched placebo controls or employ isolated components of spirulina to better understand the effects of individual bioactive compounds and elucidate their specific mechanisms of action. Another notable limitation of our study is the small, sex-imbalanced cohort, with only two female participants. This disparity limits the generalizability of our findings, especially regarding potential sex-based differences in the response to spirulina supplementation. This issue is particularly concerning given the historical underrepresentation of women in sports medicine research. Future studies should prioritize a larger, more balanced sample size, including equal representation of both sexes, to enable more robust statistical analysis and ensure the findings are applicable to both male and female athletes.

In conclusion, contradictory and inconsistent findings regarding spirulina supplementation for health and exercise performance persist in the literature. These discrepancies may arise from several factors: [1] variations in baseline nutritional status, [2] differences in training status, and [3] differing supplementation durations and dosages. The results of the current study indicate that 6 g/day of spirulina supplementation over three weeks reduced heart rate at lactate threshold during moderate simulated altitude but did not alter other physiological parameters. Additionally, it was hypothesized that the small but significant changes in red cell distribution width (RDW) may suggest the early stages of erythropoiesis, resulting in temporary variability in red blood cell size without affecting red blood cell mass. Further mechanistic research is needed to confirm these findings.

## Supplementary Material

Supplemental Material

## Data Availability

The datasets generated during and/or analyzed during the current study are available from the corresponding author on reasonable request.
