# Supplementary material for: Physiological effects of spirulina supplementation during lactate threshold exercise at simulated altitude (2,500 m): a randomized controlled trial
Source: J Int Soc Sports Nutr. 2025 May 1;22(1):2498484. doi: 10.1080/15502783.2025.2498484 (PMC12046610; doi:10.1080/15502783.2025.2498484)
Supplement: Supplemental Material [file RSSN_A_2498484_SM8291.zip › supple/Supplementary File 2.docx]

**Supplementary File 2:**

Supplementary File 2. Nutritional composition of Spirulina (100g & 6g)

|  | **Per 100g** | **Per 6g** |
| --- | --- | --- |
| **Energy** | 1420KJ/336Kcal | 85KJ/20Kcal |
| **Fat** | 1.0g | 0.05g |
| Of which saturates | 0.5g | 0.05g |
| **Carbohydrate** | 13.1g | 0.8g |
| Of which sugars | 0.0g | 0.0g |
| **Protein** | 65.9g | 3.95g |
| **Dietary Fibre** | 6.4g | 0.4g |
| **Salt** | 900.00mg | 54.000mg |
| **Vitamin A** | 0.34mg | 0.002mg |
| **Vitamin E** | 5.00mg | 0.300mg |
| **Vitamin K** | 0.03mg | 0.015mg |
| **Vitamin B1** | 2.40mg | 0.145mg |
| **Vitamin B2** | 3.70mg | 0.220mg |
| **Vitamin B3** | 12.80mg | 0.770mg |
| **Vitamin B6** | 1.40mg | 0.085mg |
| **Folate** | 0.09mg | 0.005mg |
| **Pantothenic acid** | 3.50mg | 0.210mg |
| **Potassium** | 1363.00mg | 81.78mg |
| **Calcium** | 332.50mg | 19.95mg |
| **Phosphorus** | 118.00mg | 7.80mg |
| **Magnesium** | 300.00mg | 19.00mg |
| **Iron** | 6.58mg | 0.395mg |
| **Zinc** | 2.00mg | 0.12mg |
| **Copper** | 6.10mg | 0.365mg |
| **Manganese** | 1.90mg | 0.115mg |
| **Selenium** | 0.01mg | Trace |
