# Supplementary material for: Physiological effects of spirulina supplementation during lactate threshold exercise at simulated altitude (2,500 m): a randomized controlled trial
Source: J Int Soc Sports Nutr. 2025 May 1;22(1):2498484. doi: 10.1080/15502783.2025.2498484 (PMC12046610; doi:10.1080/15502783.2025.2498484)
Supplement: Supplemental Material [file RSSN_A_2498484_SM8291.zip › supple/Supplementary File 3.docx]

Supplementary File 3.

Table 2. Test-re-Test reliability of 5 participants completing two separate LT tests at the same time of day 1-week apart at simulated altitude. HR = heart rate, RPE = Rating of Perceived Exertion, VO2 = oxygen uptake

|  | **HR**  **(b.min^-1^)** | **Power (Watts)** | **RPE** | **V̇O_2_ (ml/min/kg)** |
| --- | --- | --- | --- | --- |
| Trial 1 | 164 | 218 | 12.1 | 31.7 |
| Trial 2 | 165 | 226 | 12.2 | 32.1 |
| **Mean** | 164 | 222 | 12.2 | 31.9 |
| **SD** | 8.4 | 12 | 1.5 | 1.3 |
| **CoV%** | 1.9 | 2.9 | 5.1 | 2.7 |
| **Lower Conf. limit %** | 1.2 | 1.9 | 3.3 | 1.8 |
| **Upper Conf. limit %** | 4.5 | 7.0 | 12.5 | 6.6 |
