# Supplementary material for: Physiological effects of spirulina supplementation during lactate threshold exercise at simulated altitude (2,500 m): a randomized controlled trial
Source: J Int Soc Sports Nutr. 2025 May 1;22(1):2498484. doi: 10.1080/15502783.2025.2498484 (PMC12046610; doi:10.1080/15502783.2025.2498484)
Supplement: Supplemental Material [file RSSN_A_2498484_SM8291.zip › supple/Supplementary File 4.docx]

**Supplementary File 4:**


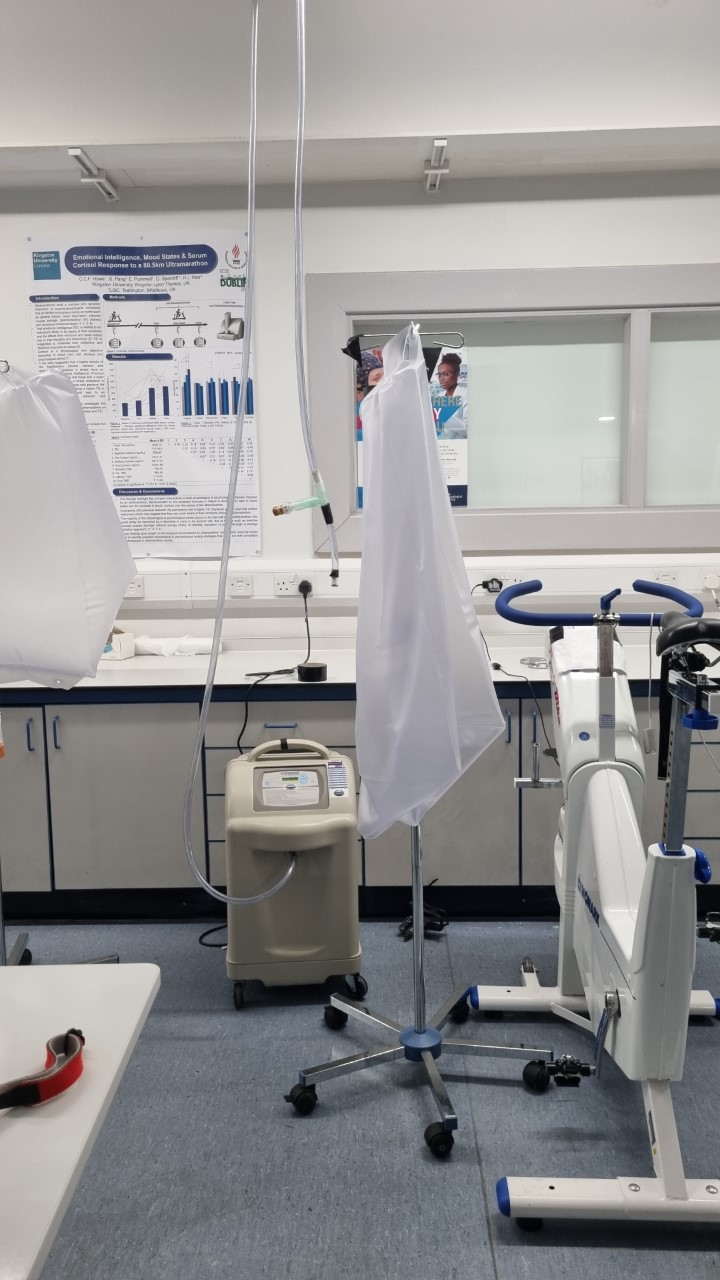


Apparatus set up. The Hypoxico generator can be seen in the bottom middle. Monark 894E Peak bike on far right. Douglas bag reservoir central.


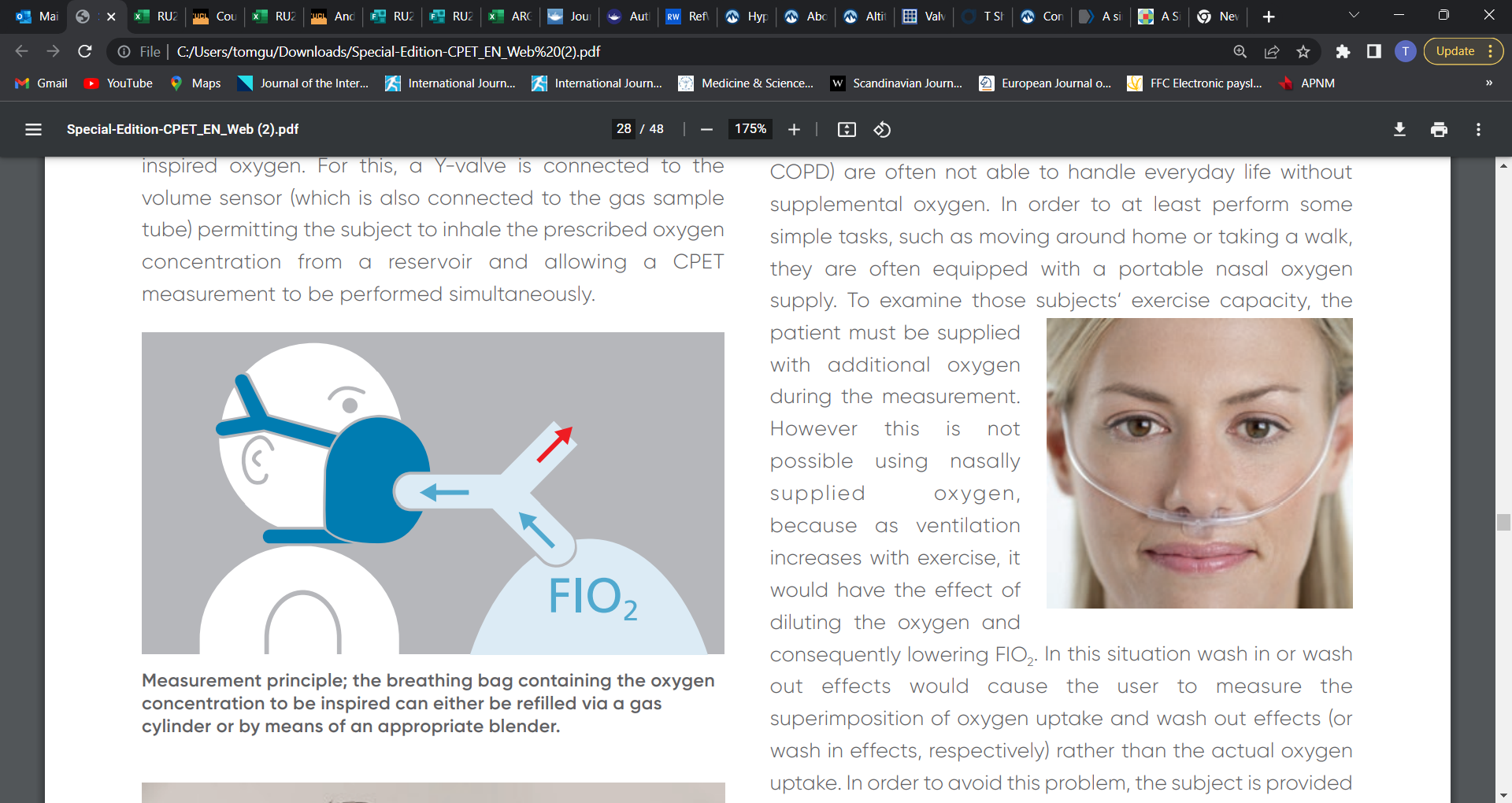

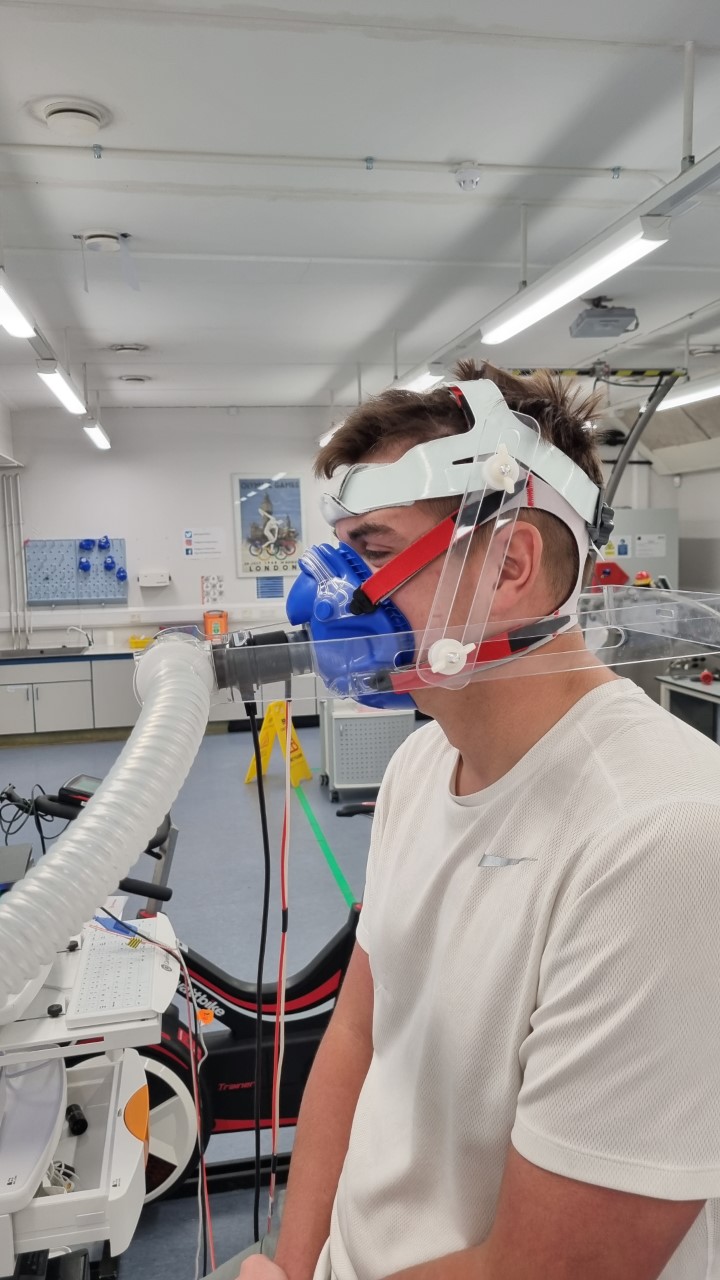


Y-Valve set up (left), Hans Rudoplh mask (blue – right), with Vyntus support helmet (far right)
