# Supplementary material for: Physiological effects of spirulina supplementation during lactate threshold exercise at simulated altitude (2,500 m): a randomized controlled trial
Source: J Int Soc Sports Nutr. 2025 May 1;22(1):2498484. doi: 10.1080/15502783.2025.2498484 (PMC12046610; doi:10.1080/15502783.2025.2498484)
Supplement: Supplemental Material [file RSSN_A_2498484_SM8291.zip › supple/Supplementary File 5.docx]

**Supplementary File 5**

Table 3. Test-re-Test reliability of 5 participants completing two separate blood tests 1-week apart. SD = standard deviation, TE = Typical Error, CV% = Coefficient of Variation %.

|  | **Test** | **Mean** | **SD** | **TE** | **CV %** | **Intraclass Correlation** | **Pearson R Correlation** |
| --- | --- | --- | --- | --- | --- | --- | --- |
| **Red Blood Cell (10^6^ /µL)** | 1  2 | 4.7  4.6 | 0.26  0.40 | 0.15 | 3.5 | 0.93 | 0.92 |
| **Haemoglobin (g/dL)** | 1  2 | 147.2  148.0 | 11.19  11.87 | 3.18 | 2.2 | 0.99 | 0.96 |
| **Hematocrit (%)** | 1  2 | 45.2  47.1 | 3.47  2.60 | 1.69 | 3.7 | 0.87 | 0.82 |
| **Mean Corpuscular Volume (µm^3^)** | 1  2 | 92.2  92.1 | 1.32  1.09 | 1.54 | 1.7 | 0.80 | 0.72 |
| **Mean Corpuscular Haemoglobin (pg)** | 1  2 | 31.8  31.7 | 0.87  0.60 | 0.42 | 1.0 | 0.96 | 0.93 |
| **Mean Corpuscular Haemoglobin Concentration (g/dL)** | 1  2 | 34.6  34.7 | 0.71  0.38 | 0.44 | 0.7 | 0.95 | 0.98 |
| **Red Cell Distribution Width (%)** | 1  2 | 12.5  12.3 | 0.68  0.49 | 0.43 | 2.1 | 0.95 | 0.91 |
| **Red Cell Distribution Width Standard Deviation (µm^3^)** | 1  2 | 42.7  42.9 | 1.09  1.35 | 0.48 | 1.4 | 0.93 | 0.86 |
| **Platelets (10^3^/µL)** | 1  2 | 220.8  225.0 | 8.76  13.02 | 0.67 | 3.3 | 0.75 | 0.69 |
| **Platelecrit (%)** | 1  2 | 0.191  0.192 | 0.003  0.005 | 0.37 | 0.8 | 0.97 | 0.96 |
| **Mean Platelet Volume (µm^3^)** | 1  2 | 8.7  8.7 | 0.17  0.18 | 1.01 | 1.5 | 0.61 | 0.55 |
| **Platelet Distribution Width (µm^3^)** | 1  2 | 13.9  13.8 | 0.3  0.1 | 1.02 | 2.1 | 0.77 | 0.88 |
| **Platelets - Large Cell Count (10^3^/µL)** | 1  2 | 56.2  55.7 | 0.7  0.6 | 1.15 | 1.4 | 0.38 | 0.45 |
| **Platelets – Large Cell Ratio (%)** | 1  2 | 25.1  25.3 | 0.6  0.4 | 0.38 | 0.9 | 0.91 | 0.97 |
| **White Blood Cells (10^3^/µL)** | 1  2 | 5.7  5.6 | 0.4  0.4 | 0.31 | 2.1 | 0.99 | 0.98 |
| **Neutrophils (10^3^/µL)** | 1  2 | 3.3  3.3 | 0.3  0.2 | 0.54 | 4.1 | 0.93 | 0.93 |
| **Lymphocytes (10^3^/µL)** | 1  2 | 1.8  1.8 | 0.2  0.2 | 0.39 | 3.5 | 0.92 | 0.98 |
| **Monocytes (10^3^/µL)** | 1  2 | 0.5  0.5 | 0.1  0.1 | 0.19 | 3.3 | 0.98 | 0.99 |
| **Eosinophils (10^3^/µL)** | 1  2 | 0.13  0.13 | 0.02  0.02 | 0.11 | 1.6 | 0.99 | 0.98 |
| **Basophils (10^3^/µL)** | 1  2 | 0.05  0.05 | 0.01  0.01 | 0.22 | 3.5 | 0.97 | 0.98 |
| **Large Immature Cells (10^3^/µL)** | 1  2 | 0.05  0.05 | 0.09  0.09 | 0.56 | 2.1 | 0.89 | 0.91 |
