# Supplementary material for: Physiological effects of spirulina supplementation during lactate threshold exercise at simulated altitude (2,500 m): a randomized controlled trial
Source: J Int Soc Sports Nutr. 2025 May 1;22(1):2498484. doi: 10.1080/15502783.2025.2498484 (PMC12046610; doi:10.1080/15502783.2025.2498484)
Supplement: Supplemental Material [file RSSN_A_2498484_SM8291.zip › supple/Supplementary File 1.doc]

**CONSORT 2010 Flow Diagram**

**Allocation**

**Analysis (N=20)**

**Follow-Up**

**Enrollment**

**Follow-Up**

Assessed for eligibility (n= 23)

Excluded (n= 3)

♦  Not meeting inclusion criteria (n=0)

♦  Declined to participate (n= 0)

♦  Other reasons (n= 3)

Lost to follow-up (give reasons) (n= 0)

Discontinued intervention (personal reasons) (n= 1)

Allocated to spirulina (n= 11)

♦ Received allocated intervention (n= 10)

Lost to follow-up (lost contact) (n= 2)

Discontinued intervention (give reasons) (n= 0)

Allocated to placebo (n= 12)

♦ Received allocated intervention (n= 10)

Randomized (n= 20)

14-day washout

Allocated to spirulina (n= 10)

♦ Received allocated intervention (n= 10)

Allocated to placebo (n= 10)

♦ Received allocated intervention (n= 10)

Lost to follow-up (give reasons)

- None

Lost to follow-up (give reasons)

- None
